# Improvement of Oil Valorization Extracted from Fish By-Products Using a Handheld near Infrared Spectrometer Coupled with Chemometrics

**DOI:** 10.3390/foods11081092

**Published:** 2022-04-10

**Authors:** Sonia Nieto-Ortega, Idoia Olabarrieta, Eduardo Saitua, Gorka Arana, Giuseppe Foti, Ángela Melado-Herreros

**Affiliations:** 1 AZTI, Food Research, Basque Research and Technology Alliance (BRTA), Parque Tecnológico de Bizkaia, Astondo Bidea, Edificio 609, 48160 Derio, Spain; iolabarrieta@azti.es (I.O.); saitua@azti.es (E.S.); gfoti@azti.es (G.F.); amelado@azti.es (Á.M.-H.); 2 Department of Analytical Chemistry, University of the Basque Country UPV/EHU, 48080 Bilbao, Spain; gorka.arana@ehu.eus

**Keywords:** no-waste, omega-3, circular economy, smart sensors, reuse, fish oil industry, recovery, chemometrics, lipid profile

## Abstract

A handheld near infrared (NIR) spectrometer was used for on-site determination of the fatty acids (FAs) composition of industrial fish oils from fish by-products. Partial least square regression (PLSR) models were developed to correlate NIR spectra with the percentage of saturated fatty acids (SFAs), monounsaturated fatty acids (MUFAs), polyunsaturated fatty acids (PUFAs) and, among them, omega-3 (ω-3) and omega-6 (ω-6) FAs. In a first step, the data were divided into calibration validation datasets, obtaining good results regarding R^2^ values, root mean square error of prediction (RMSEP) and bias. In a second step, all these data were used to create a new calibration, which was uploaded to the handheld device and tested with an external validation set in real time. Evaluation of the external test set for SFAs, MUFAs, PUFAs and ω-3 models showed promising results, with R^2^ values of 0.98, 0.97, 0.97 and 0.99; RMSEP (%) of 0.94, 1.71, 1.11 and 0.98; and bias (%) values of −0.78, −0.12, −0.80 and −0.67, respectively. However, although ω-6 models achieved a good R^2^ value (0.95), the obtained RMSEP was considered high (2.08%), and the bias was not acceptable (−1.76%). This was corrected by applying bias and slope correction (BSC), obtaining acceptable values of R^2^ (0.95), RMSEP (1.09%) and bias (−0.05%). This work goes a step further in the technology readiness level (TRL) of handheld NIR sensor solutions for the fish by-product recovery industry.

## 1. Introduction

Worldwide fisheries production and global per capita fish consumption have highly incremented in recent years [1]. The industrialization of the fish sector has brought not only a huge development but also an increase in the number of by-products generated during fish processing [2]. It is estimated that more than 70% of total fish captures are processed, of the processed products, about 50% result in solid waste and by-products [3]. These by-products are usually composed of viscera, heads, cut-offs, skin and fish that is damaged or unsuitable for human consumption [4]. Moreover, an additional source of by-products is represented by unwanted, non-targeted fish species (by-catches) that cannot be commercialized for direct human consumption [5]. These large quantities of unused fish products create serious pollution and environmental problems. Therefore, their correct reuse must become a priority for fish-processing countries and companies [6].

Most of these by-products should not be considered waste or less valuable materials [7], as they have great potential to be reused for higher-value applications [8]. Due to their high nutritive value, it is possible to give them a second life [7]. These secondary products can be processed into products such as fish sausages, pâté, cakes, gelatin, soups, sauces or snacks (i.e., the consumption of small fish bones with a minimum amount of meat as snacks, which is actually done in some countries [9]).

The production of omega-3 (ω-3)-rich fish oils represents an opportunity for valorizing fish by-products [3] and to achieve the zero-waste goal. The estimated amount of oil present in fish by-products varies from 2% to 30% of the total composition, depending on many factors, such as the fat content of the fish species and the distribution of fat in the fish body [10]. Fish oil is usually a good source of long-chain polyunsaturated fatty acids (PUFAs) [11], in particular docosahexaenoic acid (DHA) and eicosapentaenoic acid (EPA) [12]. It can be reused to generate products of high added value for the pharmaceutical industry and as raw material for food supplements [13]. Therefore, the characterization of the fatty acids (FAs) profile of fish oils is essential because EPA and DHA levels determine the destination of the product and therefore its market value [12]. Such characterization is crucial when fish oil is obtained from canning industry by-products, where it is mixed with vegetable oils, which may change the oil FAs profiles, reducing the ω-3 proportion.

Nowadays, the most common technique used to analyze the FAs profile of fish oil is gas chromatography with a flame ionization detector (GC-FID) [14], a complex technique that is relatively slow and generates toxic waste [15]. Thus, a simpler and faster technique capable of providing a response in real time would allow companies to quickly assess the FAs profile of oil and determine its most convenient destination. In this sense, near-infrared spectroscopy (NIRS) represents a valid alternative to GC-FID or other more traditional methods, as it is a rapid, non-destructive technique [16,17] that has been used in recent years in industry for quality control and process monitoring [18,19,20]. Furthermore, recent advances have allowed for a significant reduction in the size and cost of such devices, making them suitable for on-site determination [21].

This is not the first time that NIR has been used for the evaluation of the lipid profile of fish derivates. Some authors, such as Bekhit et al. and van der Merwe et al. [22,23], have studied NIR to analyze PUFAs in ω-3 supplements. Others, including dos Santos et al., analyzed the ω-3 and omega-6 (ω-6) content directly in fish fillets [24]. Other techniques, such as FT-NIR, were used by Karunathilaka et al. and Cascant et al. [14,25] to analyze omega-3 supplements and salmon. Only a few authors have used NIR to directly analyze fish oils [11] or used portable spectrometers [26]. Most research has been developed with big laboratory equipment and/or using processed fish pharmaceutical supplements or fish fillets, which prevents their use for this application in an actual industrial environment in the short term. More efforts are still needed to elevate the low technology readiness levels (TRLs) of such studies to be useful for the by-products industry. To the best of our knowledge, this is the first study demonstrating the scalability to industrial TRLs of NIR technology for measurement of the lipid profile of fish oil directly extracted from fish by-products.

Therefore, the principal objective of this study is to assess the potential of a portable device based on NIRS in combination with a partial least square regression (PLSR) analysis to characterize the FAs profile of fish oils in a rapid and non-destructive way. Thus, the device was not only calibrated to determine the ω-3 and ω-6 content but also to measure the complete fish oil profile, determining the saturated fatty acids (SFAs), monounsaturated fatty acids (MUFAs) and polyunsaturated fatty acids (PUFAs) levels. The main objective of this work was to demonstrate the high level of maturity of a handheld NIR spectroscopy sensor in combination with chemometrics for the rapid characterization of fish oil in the fish by-product industry. This technique could enable a fast and accurate classification of processed products in the appropriate market category with economic benefits for the company and increased efficiency.

## 2. Materials and Methods

### 2.1. Samples and Reagents

#### 2.1.1. Oil Mixture Preparation

Samples were supplied by a local company, which collects and reuses fishing surplus and fish industry by-products from different industrial activities. Eight fish oil samples (named with consecutive letters from A to H) obtained from fish by-products were used to make 269 different mixtures. The origin of the fish species of the oils, as well as the industries and processes from which they came, were unknown. These samples were divided in two sets: calibration (172 mixtures) and validation (97 mixtures). For external validation purposes and to ensure the robustness of the calibration, 29 new mixtures were made. The set of mixtures used for this aim was composed of three out of eight of the previous fish oils, together with a new oil (I) and two additional commercial fish oil supplements (named Supplement A and Supplement B) (Table 1).

The volume of the prepared oil mixtures was at least 3 mL. Therefore, different volumes of the initial oils were taken and mixed using automatic pipettes. The minimum amount of oil used for the mixtures was 0.1 mL, and the maximum was 2.9 mL. For some mixtures, only 2 oils were used, and the maximum number of oils used in a mixture was 6. The percentage of oil in each mixture was formulated so that the range of the mixtures covered all possible variability. All samples were filtered with Whatman grade 1 filter paper before analysis.

#### 2.1.2. Reagents

The reagents used for the methylation process of the FAs were methanol, sodium chloride, hydrochloric acid, phenolphthalein (Thermo Fisher Scientific^TM^, Roskilde, Denmark) and sodium methylate (ACROS organics^TM^, part of Thermo Fisher Scientific^TM^, Geel, Belgium). For the chromatographic analysis, n-Hexane (Thermo Fisher Scientific^TM^, Roskilde, Denmark) was used as a solvent.

### 2.2. Reference Analysis

GC-FID was employed as the reference method to analyze the fat profile of the fish oils. To extract the FAs from the oils and transform them into fatty acid methyl esters (FAMEs), the methylation process described in Commission Regulation (EC) No. 796/2002 (2002), method B, was used with some modifications [27]. In this procedure, 80 mg of sample were transferred to a flat-bottom flask, where 8 mL of sodium methylate in methanol (0.6 mol/L) and some pumice stones were added. The mixture was boiled with a reflux condenser for 10 min. Once the mixture was chilled, two drops of phenolphthalein were incorporated, and a solution of hydrochloric acid in methanol (3.5%) was added until the solution became colorless, a sign of complete acidification. The sample mixture was boiled again under the same conditions, and when cooled, 8 mL of n-hexane was added with 5 mL of a concentrate solution of sodium chloride, shaking the mixture vigorously for 1 min. Finally, the same concentrate solution of sodium chloride was added to elevate the organic phase, which contained the FAMEs, and it was transferred to a gas chromatograph vial before injection.

The solution with the FAMEs was analyzed in a gas chromatograph (Agilent 5890 from Agilent Technologies Inc., Santa Clara, CA, USA) with a DB 23 column (60 m × 0.25 mm id × 0.25 µm from J&W scientific, Santa Clara, CA, USA), a flame ionization detector (FID) and helium as carrier gas (at 30 psi and a flow rate of 1.2 mL/min). To conduct the chromatographic analysis, 2 µL of sample was injected in split mode (split of 80 mL/min) at 220 °C. The initial temperature of the chromatograph oven was 40 °C, which was maintained for 3 min. The temperature was increased at a rate of 25 °C per minute, up to 125 °C, where it was maintained for 2 min. Next, the temperature was increased again, this time at a rate of 4 °C per minute, and maintained at 180 °C for 1 min. The last temperature increase was at a rate of 1 °C per minute, up to 215 °C, where it was maintained for 10 min. Finally, the temperature of the detector was increased to 250 °C. Each GC-FID analysis was conducted 3 times.

Data from the chromatogram were collected with ChemStation Software (version A.10.02) from Agilent, (Santa Clara, CA, USA). The FAMEs of the oils, which were equal to their respective FAs, were identified with FAMEs chromatographic external standards from Sigma-Aldrich (PUFA No. 3 From Menhaden Oil and Supelco 37 Component FAME Mix). Then, the area under each FA peak was integrated in the chromatogram, and the percentage of the total oil represented by each area was calculated. Afterwards, the percentage of FAs that belonged to the same group was summed to obtain the final percentage of all the categories (SFAs, MUFAs, PUFAs, ω-3 and ω-6) for each oil. The FAs of each group are presented in Table 2.

Due to the complexity of the chromatographic method (time-consuming) and the need to analyze many samples to create a robust chemometric model, only the initial pure oils were analyzed (from A to I), and the composition of each oil mixture was calculated afterwards. To ensure that the composition of the mixtures was correct, a few were chosen randomly and analyzed.

### 2.3. NIRS Data Acquisition

A compact, handheld NIR spectrometer device was used (MicroNIR OnSite, developed by VIAVI Solutions Inc., Monza, Italy), working from 900 to 1650 nm, with a resolution of 6 nm. Samples were scanned in transflectance mode, with a special accessory for liquids (MicroNIR side-view vial holder by VIAVI) in small glass vials. A dark measurement (acquired with the lamp turned off) and a white diffuse reflectance standard (a white reference with 99% reflectance) were used for calibration. Each spectrum was the average of 100 scans, with an integration time of 8.2 ms. All spectra were taken in duplicate. For the external validation, the average of two spectra was considered for each sample.

### 2.4. Model Building

Models were developed for each FAs category: SFAs, MUFAs. PUFAs, ω-3 and ω-6. Therefore, the spectral data were considered as X, whereas the data obtained from the chromatographic analysis (the percentage of each FAs category for each model) were considered as Y.

X data were preprocessed before the multivariate analysis. Several methods were tested, such as standard normal variate without and with detrend (SNV and SNVd), multiplicative scatter correction (MSC), Saviztky–Golay first and second derivatives (with different polynomial orders and windows) and combinations of all of them. This step was necessary because it eliminates the irrelevant information that cannot be correctly processed [28], and it improves the regression [29]. X and Y data were mean centered in all cases before creation of the models.

To correlate the NIR spectra and the reference data (SFAs, MUFAs, PUFAs, ω-3 and ω-6 percentage), several partial least square regression (PLSR) models were developed [30]. For each developed model, two steps were followed:

- 1st step: The five models were built using Matlab R2013a equipped with the PLS_toolbox (version 8.2.1) (The Mathworks, Natick, USA). For the calibration, *n*_c.1_ = 172 samples were used, and one model was developed for each FAs category. In all cases, a venetian blinds cross-validation (CV) with 10 data splits and 2 samples per blind was carried out. Then, the model was validated using the validation set (*n*_v.1_ = 97).
- 2nd step: All the data used in the previously developed models (*n*_c.1_ and *n*_v.1_) were used to create a new dataset, which was used as calibration dataset (*n*_c.2_ = 269). Then, a random CV with 20 segments and 27 samples per segment was carried out. These models were uploaded into the MicroNIR OnSite to directly predict an external dataset (*n*_test_ = 29) in real time in the place of analysis and without the necessity of extracting the data from the spectrometer and analyzing it afterwards in a computer. To build the mentioned calibration model, The Unscrambler^®^ X 10.5.1 software was used (CAMO Software AS, Oslo, Norway).

As figures of merit of the models, the coefficient of determination (R^2^), the root mean square error (RMSE) and the bias value were calculated for the CV and the prediction. To study the distribution of the oil mixtures used in each dataset, their mean, standard deviation, minimum and maximum values were calculated and expressed in percentage.

## 3. Results and Discussion

### 3.1. Determination of Fatty Acid Profiles of the Samples by Reference Analysis

#### 3.1.1. Fatty Acid Composition of the Initial Oils

Table 3 displays the composition (% of the FAs categories) of the nine initial oils from which the mixtures were made and the two commercial supplements of ω-3.

Oil samples show high variability in their FAs profiles. This suggests that the oils collected for this study might have different origins and could come from different kinds of fish, production methods or various types of processing industries. This sample variability highlights the importance of determining the lipid profile of fish oils, as the percentage of the different groups, especially ω-3, varies significantly between samples.

On the one hand, oils A, C and F and Supplement B showed the typical seawater fish oil composition regarding PUFAs, where most of the PUFAs come from ω-3 FAs [31,32,33]. In these samples, PUFAs represented between 30% and 46% of the total FAs of the oils. They had an elevated ω-3 content, which almost corresponded with all the PUFAs in the samples, and a lower content of ω-6. Considering their composition, these samples may come from a process where only seawater fish is involved, i.e., fish fillet processing [31,32].

On the other hand, samples B, D, E, G, H and I and supplement A had PUFAs content between 25% and 34%, which is also typical in fish oil [31]. However, these samples presented a higher content of ω-6 FAs than the previous set, in which ω-3 FAs were predominant. This is due to a high level of linoleic acid (18:2) (data not shown), which may have two explanations: on the one hand, it might be due to the fish species from which the oil was obtained, i.e., this PUFAs profile is characteristic of freshwater fish [34], which has a higher ω-6 content in comparison with seawater fish [32,33]. On the other hand, it might be due to the type of fish processing industry from which the samples originated. In the canning industry, by-products of fish oil are mixed with vegetable oils, such as sunflower oil, which is rich in ω-6 PUFAs (linoleic acid) [35,36].

#### 3.1.2. Fatty Acid Profile of the Oil Mixtures

The results of the characterization and the statistics of the oil samples in the different sets of data used in the models are shown in Table 4.

MUFAs constitute the majority group in most cases representing: in the 1st step, 41.4% of total FAs composition on the calibration set and, in the 2nd step, the 40.1% of the calibration set and the 43.7% of the external validation set. However, PUFAs are the majority group in the validation set of the 1st step, with a percentage of 38.6%. On the contrary, ω-6 is the least common group in the four sets of samples, with percentages of 10.3% and 8.6% in the calibration and validation set of the first step, respectively, and 9.8% and 12.1% in the calibration and external validation set of the second step, respectively.

### 3.2. Performance of the PLSR Models of the Target Oils

#### 3.2.1. Model Results

The CV and validation results of the five models developed for each category of FAs in the first step are shown in Table 5.

In Table 5, all the models developed for the validation of SFAs, MUFAs, PUFAs, ω-3 and ω-6 achieved good results, with R^2^_val_ values of 0.98, 0.97, 0.96, 0.99 and 0.95; small errors of 0.68, 1.27, 0.85, 0.60 and 0.90; and a low bias value of −0.40, 0.25, −0.49, −0.26, −0.34, respectively.

On the other hand, the results of the five models developed in the second step (CV and external validation) are shown in Table 6.

In this case (Table 6), models for SFAs, MUFAs, PUFAs and ω-3 achieved good results in the external validation set regarding R^2^ (0.98, 0.97, 0.97 and 0.99), RMSEP (0.94%, 1.71%, 1.11% and 0.98%) and bias (−0.78%, −0.12%, −0.80% and −0.67%), respectively.

**Figure 1:**
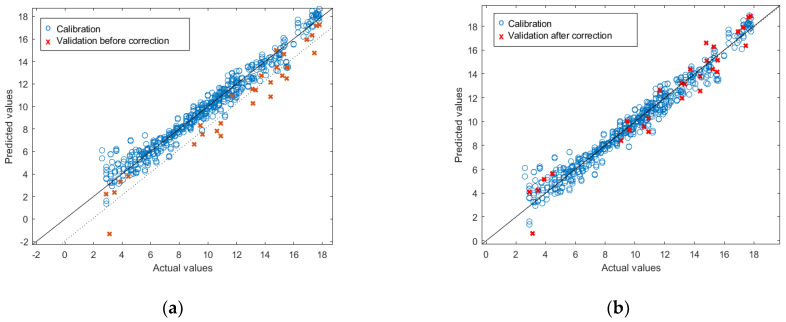
Results of the external validation of the PLSR for prediction of ω-6 before (**a**) and after (**b**) bias and slope correction.

Although the ω-6 model achieved good results in terms of R^2^, the RMSEP and the bias in the validation showed high values: 2.09% and −1.76%, respectively. This is very common in quantitative NIRS and may be due to block effects occurring between measuring conditions [37]. In this case, there are two possible reasons for these effects. (i) The measurement conditions: all the measurements were performed in a laboratory under controlled temperature; therefore, the authors believe they might have a small effect. (ii) The possibly different origins of the oils, including different fish species and different processing industries. Seawater fish, the most consumed type of fish, is naturally low in ω-6 FAs, with most PUFAs resulting from the presence of ω-3 FAs [38,39]. However, as stated in Section 3.1.1, some of the fish oil samples had a higher content of ω-6 FAs. This finding could result from: (i) the presence of vegetable oils mixed with the fish oil, which is plausible if some of the samples came from the canning industry or (ii) the presence of samples from industries where the raw material is freshwater fish. However, the model can be corrected using techniques such as bias and slope correction (BSC) [40]. Applying this technique to the external test set (Figure 1), the following results are obtained: R^2^ = 0.95; RMSEP = 1.09%; bias = −0.05%.

These results are in accordance with those of other studies found in the literature that studied the fish oil profile of different matrices. In dietary supplements, Hespanhol et al. [26] and Bekhit et al. [22] obtained similar R^2^ values (0.97 and 0.98, respectively) for ω-3 prediction, although their models were less complex, with one and two latent variables (LVs), respectively. The differences in complexity may be due to the fact that in the present study, the fish oil was analyzed directly from by-products with no previous processing (cleaning, refining, etc.), as it was made with dietary supplements. The results from the MUFAs, ω-3 and ω-6 models are similar to those obtained by Karunathilaka et al. [14] in dietary supplements, with RMSEP values of 1.03, 1.42 and 0.93, respectively. In other matrices, such as the model system created by Afseth et al. (using 70 different mixtures of protein, water and oil blends) [41], the error obtained for SFAs, MUFAs and PUFAs was similar to our results, with RMSEP values of 1.20, 0.80 and 0.60, respectively.

The good results achieved by the SFAs, MUFAs, PUFAs and ω-3 models in external validation and in the ω-6 models after the BSC suggest that the models can predict new samples from different fish oil industries. Furthermore, the ω-6 model could be improved with the addition of new samples of different origins, which would correct the bias and slope deviation.

#### 3.2.2. Spectral Information of the Models

**Figure 2:**
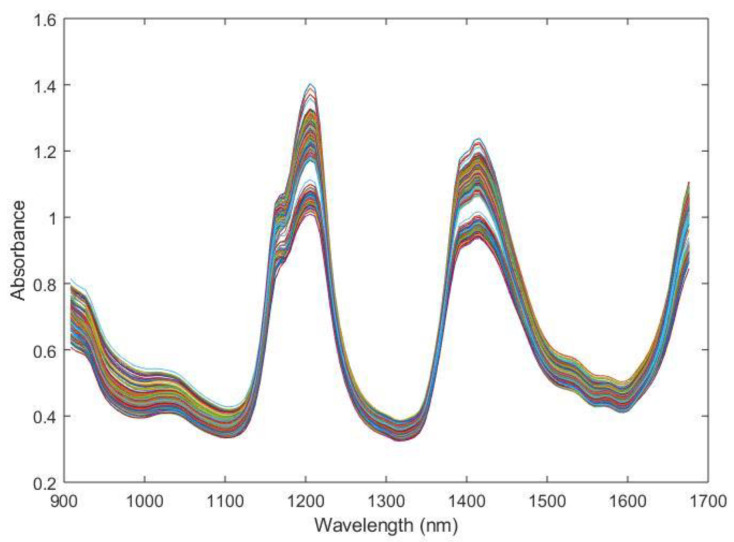
Raw spectra of the oil mixtures used during the experiment.

Raw spectra of the oil mixtures used during the experiment are shown in Figure 2.

Although information is usually hidden in the NIR spectrum, characteristic absorption bands from oil samples are observed in the raw spectra (Figure 2) at 900, 1020, 1200 and 1400 nm. The first two weak peaks observed are around 900 and 1020 nm. The former corresponds to the C-H stretching third overtone of CH_3_, whereas the latter is a combination of the C-H stretching first overtone and the C-H deformation second overtone, again from CH_3_ [11]. The first strong peak at 1200 nm is due to the second overtone of the stretching mode of C-H bonds in various chemical groups [42,43]. The second strong peak, localized between 1300 and 1500 nm, is caused by the combination of the stretching and deformation first overtone of C-H in CH, CH_2_ and CH_3_ [11].

**Figure 3:**
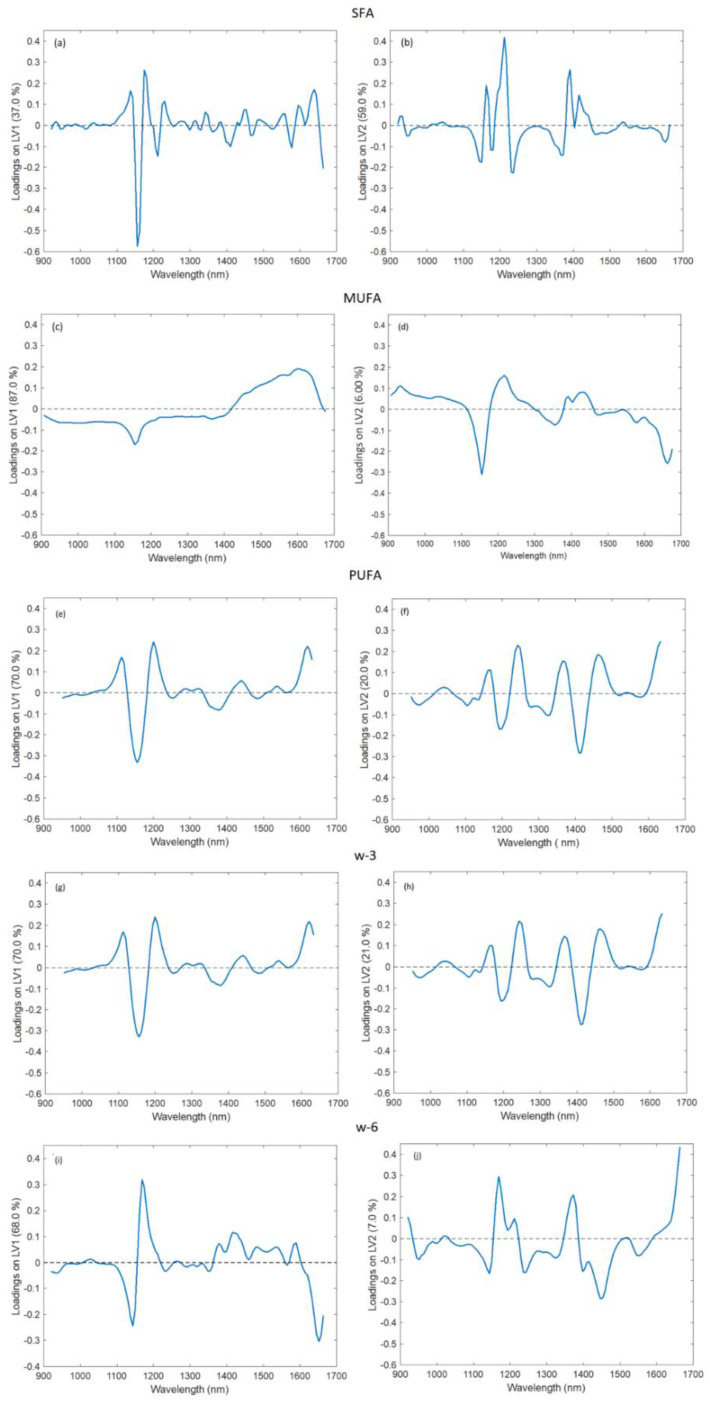
LV1 and LV2 of the second-step models. (**a**,**b**) SFAs, (**c**,**d**) MUFAs, (**e**,**f**) PUFAs, (**g**,**h**) ω-3, (**i**,**j**) ω-6.

The loadings corresponding to the first and second latent variables (LV1 and LV2) of the five models developed in the second step, which contain information about all the data used in the experiments, are shown in Figure 3. LV1 retains the greatest amount of variance in most of the models, except for the SFAs model, wherein LV2 retains the most information. The large peaks in the loadings of the models resemble the main peaks of the raw spectra.

NIR absorption peaks related to the FAs information are associated with the vibrations of C-H and CH_2_ [44]. Although they are usually above 1700 nm in the spectra, where two important regions are located at 1720 and 2143 nm [45], the presence of other bands related to C-H overtones at shorter wavelengths makes possible the measurement of oils with devices whose spectral range covers only wavelengths lower than 1700 nm, as demonstrated by Basri et al. [46].

As can be seen in Figure 3a–e, LV1 and LV2 of all the models show important peaks in the region between 1050 and 1300 nm. This region corresponds to the second overtone of C-H stretching, and it is one of the most important regions to determine FAs with this technology [42,43,44].

LV1 of PUFAs, ω-3 and ω-6 (Figure 3c–e) and LV2 in all the models (Figure 3a–e) show peaks in the region between 1300 and 1500 nm (Figure 3a,c–e). This absorption region is caused by the combination of the stretching and deformation of the first overtone of C-H in CH, CH_2_ and CH_3_ [11].

The increase found in the region between 1600 and 1670 nm can be seen in LV1 of PUFAs, ω-3 and ω-6 (Figure 3c–e) and in LV2 of MUFAs, PUFAs, ω-3 and ω-6 (Figure 3b–e). According to Hourant et al. [47], the wavelengths between 1600 and 1780 nm are related to the first overtone of the C-H group in -CH_3_, and the peak that is starting to grow may correspond with the first part of that region. On the contrary, LV1 of SFAs and MUFAs (Figure 3a,b) and LV2 of SFAs (Figure 3a) present a peak with a maximum around 1600 nm. This region of the spectra is related to the C-H first overtone of = CH_2_, which acquires its maximum at 1620 nm [48].

The similarity in shape between PUFAs and ω-3 loadings suggests that they are closely related (Figure 3c,d). ω-6 loadings also present peaks at similar wavelengths (Figure 3e) as PUFAs and ω-3 loadings. This result was expected because fish PUFAs are mostly composed of ω-3 and ω-6 FAs [49], as can be seen in Table 3.

## 4. Conclusions

This study demonstrates the possibility of using a handheld NIR spectrometer as an alternative to GC-FID to determine fish oil fat composition on-site in a fast and non-destructive way. NIR spectroscopy, coupled with chemometrics, can predict concentrations of SFAs, MUFAs, PUFAs and ω-3 FAs with good results, with the SFAs and ω-3 models performing best in external validation (R^2^ of 0.98 and 0.99, RMSEP = 0.94% and 0.98%, and BIAS = 0.78% and −0.67%, respectively, in the test set).

Although the technique produced a high error of prediction and bias in the ω-6 FAs model (RMSEP = 2.09% and Bias = −1.76%), this was corrected with the application of BSC, obtaining an R^2^ of 0.95, an RMSEP of 1.09% and a bias of −0.05%, which could be improved in the future with the addition of new oil samples to the model.

The results presented in this study demonstrate that NIR spectroscopy is a mature technology capable of rapidly and efficiently determining the quality of oils extracted from fish by-products, which makes it suitable for industrial applications. This will allow food industries to rapidly and efficiently determine the quality and commercial value of oil coming from fish by-products.

## Figures and Tables

**Table 1:** Samples of oil mixtures used in each dataset.

	Calibration Set	Validation Set	External Validation Set
Number of mixtures	172	97	29
Oils and supplements used	A, B, C, D, E, F and G	B, E, F, G and H	B, E, F, I, Supplement A and Supplement B

**Table 2:** Identified FAs in each category.

FAs Group	Fatty Acids
SFAs	Myristic (14:0), Palmitic (16:0), Stearic (18:0), Arachidic (20:0)
MUFAs	Palmitoleic (16:1), Oleic (18:1), Gadoleic (20:1), Erucic (22:1)
PUFAs	Linoleic (18:2), Gamma-linolenic (18:3), Stearidonic (18:4), Arachidonic (20:4), EPA (20:5), Clupanodonic (22:5), DHA (22:6).
ω-3	Alpha-linoleic (18:3), Stearidonic (18:4), EPA (20:5), Clupanodonic (22:5), DHA (22:6)
ω-6	Linoleic (18:2), Arachidonic (20:4)

SFAs: saturated fatty acids, MUFAs: monounsaturated fatty acids, PUFAs: polyunsaturated fatty acids, ω-3: omega-3 fatty acids, ω-6: omega-6 fatty acids.

**Table 3:** Composition (%) of the initial oils with their standard deviation.

Oils	SFAs	MUFAs	PUFAs	ω-3	ω-6
A	27.84 ± 0.32	42.05 ± 0.08	30.11 ± 0.25	27.50 ± 0.23	2.61 ± 0.03
B	14.26 ± 0.08	53.76 ± 0.12	31.98 ± 0.05	14.18 ± 0.05	17.80 ± 0.02
C	29.27 ± 0.30	25.60 ± 0.14	45.13 ± 0.30	42.24 ± 0.31	2.90 ± 0.00
D	17.47 ± 0.19	49.26 ± 0.03	33.27 ± 0.21	17.70 ± 0.13	15.57 ± 0.09
E	21.09 ± 0.26	44.94 ± 0.52	33.98 ± 0.30	22.27 ± 0.10	11.70 ± 0.32
F	29.66 ± 0.19	25.58 ± 0.09	44.76 ± 0.15	41.88 ± 0.14	2.89 ± 0.02
G	17.51 ± 0.21	49.10 ± 0.16	33.39 ± 0.07	17.87 ± 0.05	15.52 ± 0.03
H	18.51 ± 0.40	48.75 ± 0.19	32.74 ± 0.22	17.43 ± 0.14	15.31 ± 0.07
I	18.10 ± 0.08	49.18 ± 0.31	32.73 ± 0.24	17.50 ± 0.08	15.23 ± 0.17
Supplement A	14.34 ± 0.02	56.56 ± 0.30	29.10 ± 0.32	14.25 ± 0.39	14.85 ± 0.08
Supplement B	29.50 ± 0.02	25.32 ± 0.03	45.19 ± 0.05	42.08 ± 0.07	3.11 ± 0.01

For each sample, three replications were performed. SFAs: saturated fatty acids, MUFAs: monounsaturated fatty acids, PUFAs: polyunsaturated fatty acids, ω-3: omega-3 fatty acids, ω-6: omega-6 fatty acids.

**Table 4:** Results of the characterization of all the samples.

		Dataset	*n*	Mean ± SD (%)	Minimum (%)	Maximum (%)
SFAs	1st Step	Calibration	172	22.0 ± 3.8	14.3	29.7
		Validation	97	24.1 ± 3.8	14.4	29.7
	2nd Step	Calibration	269	22.7 ± 3.8	14.3	29.7
		External Validation	29	20.5 ± 4.8	14.3	29.7
MUFAs	1st Step	Calibration	172	41.4 ± 6.9	25.6	53.8
		Validation	97	37.2 ± 7.4	25.6	53.6
	2nd Step	Calibration	269	40.1 ± 7.1	25.6	53.8
		External Validation	29	43.7 ± 9.2	25.3	56.6
PUFAs	1st Step	Calibration	172	36.5 ± 3.6	30.1	45.1
		Validation	97	38.6 ± 3.6	32.0	44.8
	2nd Step	Calibration	269	37.2 ± 3.6	30.1	45.1
		External Validation	29	35.8 ± 4.4	29.1	45.2
ω-3	1st Step	Calibration	172	26.2 ± 6.9	14.2	42.2
		Validation	97	30.0 ± 7.4	14.3	41.9
	2nd Step	Calibration	269	27.4 ± 7.0	14.2	42.2
		External Validation	29	23.7 ± 9.0	14.2	42.1
ω-6	1st Step	Calibration	172	10.3 ± 3.8	2.6	17.8
		Validation	97	8.6 ± 3.9	2.9	17.7
	2nd Step	Calibration	269	9.8 ± 3.8	2.6	17.8
		External Validation	29	12.1 ± 4.7	2.9	17.8

*n*: number of samples, SD: standard deviation, SFAs: saturated fatty acids, MUFAs: monounsaturated fatty acids, PUFAs: polyunsaturated fatty acids, ω-3: omega-3 fatty acids, ω-6: omega-6 fatty acids.

**Table 5:** Principal statistics of the five models developed in the first step.

		X Preprocessing	Y Preprocessing	LV	R2	RMSE (%)	Bias (%)
SFAs	CV	2nd derivative (order 2, window 5) + Mean Center	Mean Center	5	0.98	0.57	−2 × 10^−3^
	Validation				0.98	0.68	−0.40
MUFAs	CV	SNV + Mean Center	Mean Center	3	0.99	0.74	−3 × 10^−4^
	Validation				0.97	1.27	0.25
PUFAs	CV	SNV + 2nd derivative (order 2, window 15) + Mean Center	Mean Center	5	0.97	0.65	2 × 10^−4^
	Validation				0.96	0.85	−0.49
ω-3	CV	SNV + 2nd derivative (order 2, window 15) + Mean Center	Mean Center	6	0.99	0.48	−2 × 10^−3^
	Validation				0.99	0.60	−0.26
ω-6	CV	MSC (using the mean of the spectra as reference) + 1st derivative (order 2 window 5) + Mean Center	Mean Center	6	0.96	0.78	0.02
	Validation				0.95	0.90	−0.34

LV: latent variables, CV: cross-validation, SNV: standard normal variate, MSC: multiplicative scatter correction, RMSE: root mean square error, SFAs: saturated fatty acids, MUFAs: monounsaturated fatty acids, PUFAs: polyunsaturated fatty acids, ω-3: omega-3 fatty acids, ω-6: omega-6 fatty acids.

**Table 6:** Principal statistics calculated for the five models developed in the second step.

		X Pretreatment	Y Pretreatment	LV	R2	RMSE (%)	Bias (%)
SFAs	CV	2nd derivative (order 2, window 5) + Mean Center	Mean Center	5	0.98	0.60	−4 × 10^−3^
	External validation				0.98	0.94	−0.78
MUFAs	CV	SNV + Mean Center	Mean Center	3	0.99	0.77	5 × 10^−4^
	External validation				0.97	1.71	−0.12
PUFAs	CV	SNV + 2nd derivative (order 2, window 15) + Mean Center	Mean Center	5	0.97	0.65	2 × 10^−3^
	External validation				0.97	1.11	−0.80
ω-3	CV	SNV + 2nd derivative (order 2, window 15) + Mean Center	Mean Center	6	0.99	0.71	−5 × 10^−6^
	External validation				0.99	0.98	−0.67
ω-6	CV	MSC (using the mean of the spectra as reference) + 1st derivative (order 2 window 5) + Mean Center	Mean Center	6	0.96	0.74	−1 × 10^−4^
	External validation				0.95	2.09	−1.76

LV: latent variables, CV: cross-validation, SNV: standard normal variate, MSC: multiplicative scatter correction, RMSE: root mean square error, SFAs: saturated fatty acids, MUFAs: monounsaturated fatty acids, PUFAs: polyunsaturated fatty acids, ω-3: omega-3 fatty acids, ω-6: omega-6 fatty acids.

## Data Availability

The datasets generated for this study are available on request from the corresponding author.

## References

[1] Nawaz A., Li E., Irshad S., Hhm H., Liu J., Shahbaz H.M., Ahmed W., Regenstein J.M. (2020). Improved effect of autoclave processing on size reduction, chemical structure, nutritional, mechanical and in vitro digestibility properties of fish bone powder. Adv. Powder Technol..

[2] Gehring C.K., Gigliotti J.C., Moritz J.S., Tou J.C., Jaczynski J. (2011). Functional and nutritional characteristics of proteins and lipids recovered by isoelectric processing of fish by-products and low-value fish: A review. Food Chem..

[3] Rubio-Rodríguez N., de Diego S.M., Beltrán S., Jaime I., Sanz M.T., Rovira J. (2012). Supercritical fluid extraction of fish oil from fish by-products: A comparison with other extraction methods. J. Food Eng..

[4] Rustad T., Storrø I., Slizyte R. (2011). Possibilities for the utilisation of marine by-products. Int. J. Food Sci. Technol..

[5] Iñarra B., Bald C., Cebrián M., Peral I., Llorente R., Zufía J. (2020). Evaluation of unavoidable unwanted catches valorisation options: The Bay of Biscay case study. Mar. Policy.

[6] Rodriguez Y.E., Pereira N.A., Haran N.S., Mallo J.C., Fernandez-Gimenez A.V. (2017). A new approach to fishery waste revalorization to enhance Nile tilapia (*Oreochromis niloticus*) digestion process. Aquac. Nutr..

[7] Simat V., Vlahovic J., Soldo B., Skroza D., Ljubenkov I., Mekinic I.G. (2019). Production and Refinement of Omega-3 Rich Oils from Processing By-Products of Farmed Fish Species. Foods.

[8] Aspevik T., Oterhals Å., Rønning S.B., Altintzoglou T., Wubshet S.G., Gildberg A., Afseth N.K., Whitaker R.D., Lindberg D., Lin C.S.K. (2018). Valorization of Proteins from Co- and By-Products from the Fish and Meat Industry. Chemistry and Chemical Technologies in Waste Valorization.

[9] FAO (2020). The State of World Fisheries and Aquaculture. https://www.fao.org/3/ca9229en/ca9229en.pdf.

[10] Ramakrishnan V.V., Ghaly A.E., Brooks M.S., Budge S.M. (2013). Extraction of oil from mackerel fish processing waste using Alcalase Enzyme. Enzym. Eng..

[11] Wu D., Chen X.J., Cao F., Sun D.W., He Y., Jiang Y.H. (2014). Comparison of Infrared Spectroscopy and Nuclear Magnetic Resonance Techniques in Tandem with Multivariable Selection for Rapid Determination of omega-3 Polyunsaturated Fatty Acids in Fish Oil. Food Bioprocess Technol..

[12] Cheng J.H., Sun D.W., Liu G.X., Chen Y.N. (2019). Developing a multispectral model for detection of docosahexaenoic acid (DHA) and eicosapentaenoic acid (EPA) changes in fish fillet using physarum network and genetic algorithm (PN-GA) method. Food Chem..

[13] Al Khawli F., Pateiro M., Dominguez R., Lorenzo J.M., Gullon P., Kousoulaki K., Ferrer E., Berrada H., Barba F.J. (2019). Innovative Green Technologies of Intensification for Valorization of Seafood and Their By-Products. Mar. Drugs.

[14] Karunathilaka S.R., Choi S.H., Mossoba M.M., Yakes B.J., Bruckner L., Ellsworth Z., Srigley C.T. (2019). Rapid classification and quantification of marine oil omega-3 supplements using ATR-FTIR, FT-NIR and chemometrics. J. Food Compos. Anal..

[15] Hernandez-Martinez M., Gallardo-Velazquez T., Osorio-Revilla G., Almaraz-Abarca N., Ponce-Mendoza A., Vasquez-Murrieta M.S. (2013). Prediction of total fat, fatty acid composition and nutritional parameters in fish fillets using MID-FTIR spectroscopy and chemometrics. LWT Food Sci. Technol..

[16] Alexandrakis D., Downey G., Scannell A.G.M. (2012). Rapid Non-destructive Detection of Spoilage of Intact Chicken Breast Muscle Using Near-infrared and Fourier Transform Mid-infrared Spectroscopy and Multivariate Statistics. Food Bioprocess Technol..

[17] Leme L.M., Nakamura F., Tanamati A.A.C., Valderrama P., Marco P.H. (2019). Fast non-invasive screening to detect fraud in oil capsules. LWT Food Sci. Technol..

[18] Siesler H.W., Burns D.A., Ciurczak E.W. (2008). Basic Principles of Near-Infrared Spectroscopy. Handbook of Near-Infrared Analysis.

[19] Salguero-Chaparro L., Baeten V., Fernández-Pierna J.A., Peña-Rodríguez F. (2013). Near infrared spectroscopy (NIRS) for on-line determination of quality parameters in intact olives. Food Chem..

[20] Ríos-Reina R., García-González D.L., Callejón R.M., Amigo J.M. (2018). NIR spectroscopy and chemometrics for the typification of Spanish wine vinegars with a protected designation of origin. Food Control.

[21] Marques E.J.N., de Freitas S.T., Pimentel M.F., Pasquini C. (2016). Rapid and non-destructive determination of quality parameters in the ‘Tommy Atkins’ mango using a novel handheld near infrared spectrometer. Food Chem..

[22] Bekhit M.Y., Grung B., Mjøs S.A. (2014). Determination of Omega-3 Fatty Acids in Fish Oil Supplements Using Vibrational Spectroscopy and Chemometric Methods. Appl. Spectrosc..

[23] Van der Merwe S., Manley M., Wicht M. (2018). Enhancing near infrared spectroscopy models to identify omega-3 fish oils used in the nutraceutical industry by means of calibration range extension. J. Near Infrared Spectrosc..

[24] Dos Santos D.A., Coqueiro A., Gonçalves T.R., Carvalho J.C., Bezerra J.S., Matsushita M., de Oliveira C.A.L., Março P.H., Valderrama P., Ribeiro R.P. (2020). Omega-3 and Omega-6 Determination in Nile Tilapia’s Fillet Based on MicroNIR Spectroscopy and Multivariate Calibration. J. Braz. Chem. Soc..

[25] Cascant M.M., Breil C., Fabiano-Tixier A.S., Chemat F., Garrigues S., de la Guardia M. (2018). Determination of fatty acids and lipid classes in salmon oil by near infrared spectroscopy. Food Chem..

[26] Hespanhol M.C., Souza J.C., Pasquini C. (2020). Feasibility of a portable, low-cost near-infrared spectrophotometer for the quality screening of omega-3 dietary supplements. J. Pharm. Biomed. Anal..

[27] EUR-lex Commission Regulation (EC) No 796/2002. https://eur-lex.europa.eu/eli/reg/2002/796/oj.

[28] Nicolai B.M., Beullens K., Bobelyn E., Peirs A., Saeys W., Theron K.I., Lammertyn J. (2007). Nondestructive measurement of fruit and vegetable quality by means of NIR spectroscopy: A review. Postharvest Biol. Technol..

[29] Rinnan Å., van den Berg F., Engelsen S.B. (2009). Review of the most common pre-processing techniques for near-infrared spectra. TrAC Trends Anal. Chem..

[30] Wold S., Sjöström M., Eriksson L. (2001). PLS-regression: A basic tool of chemometrics. Chemom. Intell. Lab. Syst..

[31] Özogul Y., Özogul F. (2007). Fatty acid profiles of commercially important fish species from the Mediterranean, Aegean and Black Seas. Food Chem..

[32] Özogul Y., Özogul F., Alagoz S. (2007). Fatty acid profiles and fat contents of commercially important seawater and freshwater fish species of Turkey: A comparative study. Food Chem..

[33] Li G., Sinclair A.J., Li D. (2011). Comparison of Lipid Content and Fatty Acid Composition in the Edible Meat of Wild and Cultured Freshwater and Marine Fish and Shrimps from China. J. Agric. Food Chem..

[34] Rahnan S.A., Huah T.S., Nassan O., Daud N.M. (1995). Fatty acid composition of some Malaysian freshwater fish. Food Chem..

[35] Moret S., Purcaro G., Conte L.S. (2005). Polycyclic aromatic hydrocarbons in vegetable oils from canned foods. Eur. J. Lipid Sci. Technol..

[36] Ganesan K., Sukalingam K., Xu B. (2018). Impact of consumption and cooking manners of vegetable oils on cardiovascular diseases—A critical review. Trends Food Sci. Technol..

[37] Melado-Herreros A., Nieto-Ortega S., Olabarrieta I., Gutiérrez M., Villar A., Zufía J., Gorretta N., Roger J.-M. (2021). Postharvest ripeness assessment of ‘Hass’ avocado based on development of a new ripening index and Vis-NIR spectroscopy. Postharvest Biol. Technol..

[38] Özogul Y., Özogul F., Çïçek E., Polat A., Kuley E. (2009). Fat content and fatty acid compositions of 34 marine water fish species from the Mediterranean Sea. Int. J. Food Sci. Nutr..

[39] Kocatepe D., Turan H. (2012). Proximate and Fatty Acid Composition of Some Commercially Important Fish Species from the Sinop Region of the Black Sea. Lipids.

[40] Osborne B.G., Fearn T. (1983). Collaborative evaluation of universal calibrations for the measurement of protein and moisture in flour by near infrared reflectance. Int. J. Food Sci. Technol..

[41] Afseth N.K., Segtnan V.H., Marquardt B.J., Wold J.P. (2005). Raman and near-infrared spectroscopy for quantification of fat composition in a complex food model system. Appl. Spectrosc..

[42] Downey G., McIntyre P., Davies A.N. (2002). Detecting and Quantifying Sunflower Oil Adulteration in Extra Virgin Olive Oils from the Eastern Mediterranean by Visible and Near-Infrared Spectroscopy. J. Agric. Food Chem..

[43] Garrido-Varo A., Sánchez M.-T., De la Haba M.-J., Torres I., Pérez-Marín D. (2017). Fast, low-cost and non-destructive physico-chemical analysis of virgin olive oils using near-infrared reflectance spectroscopy. Sensors.

[44] Cheng J.H., Sun D.W. (2017). Partial Least Squares Regression (PLSR) Applied to NIR and HSI Spectral Data Modeling to Predict Chemical Properties of Fish Muscle. Food Eng. Rev..

[45] Martín J.F.G. (2015). Optical path length and wavelength selection using Vis/NIR spectroscopy for olive oil’s free acidity determination. Int. J. Food Sci. Technol..

[46] Basri K.N., Hussain M.N., Bakar J., Sharif Z., Khir M.F.A., Zoolfakar A.S. (2017). Classification and quantification of palm oil adulteration via portable NIR spectroscopy. Spectrochim. Acta Part A.

[47] Hourant P., Baeten V., Morales M.T., Meurens M., Aparicio R. (2000). Oil and Fat Classification by Selected Bands of Near-Infrared Spectroscopy. Appl. Spectrosc..

[48] Shenk J.S., Workman J.J., Westerhaus M.O., Burns D.A., Ciurczak E.W. (2008). Application of NIR spectroscopy to agricultural products. Handbook of Near-Infrared Analysis.

[49] Aubourg S.P., Nollet L.M.L., Toldrá F. (2010). Lipid compounds. Hanbook of Seafood and Seafood Products Analysis.

